# Progress of Electrospun Nanofibrous Carriers for Modifications to Drug Release Profiles

**DOI:** 10.3390/jfb13040289

**Published:** 2022-12-09

**Authors:** Ying Wang, Deng-Guang Yu, Yang Liu, Ya-Nan Liu

**Affiliations:** 1 School of Materials and Chemistry, University of Shanghai for Science and Technology, Shanghai 200093, China; 2 Shanghai Engineering Technology Research Center for High-Performance Medical Device Materials, Shanghai 200093, China; 3 School of Chemistry and Chemical Engineering, Shanghai University of Engineering Science, 333 Long Teng Road, Shanghai 201620, China

**Keywords:** electrospinning, medicated nanofibers, drug delivery, controlled release, functional biomaterials

## Abstract

Electrospinning is an advanced technology for the preparation of drug-carrying nanofibers that has demonstrated great advantages in the biomedical field. Electrospun nanofiber membranes are widely used in the field of drug administration due to their advantages such as their large specific surface area and similarity to the extracellular matrix. Different electrospinning technologies can be used to prepare nanofibers of different structures, such as those with a monolithic structure, a core–shell structure, a Janus structure, or a porous structure. It is also possible to prepare nanofibers with different controlled-release functions, such as sustained release, delayed release, biphasic release, and targeted release. This paper elaborates on the preparation of drug-loaded nanofibers using various electrospinning technologies and concludes the mechanisms behind the controlled release of drugs.

## 1. Introduction

Because the body’s self-healing function is limited, when the human body is injured, it is often necessary to use drugs to speed up the repair process. Drugs can inhibit internal and external factors that are not conducive to human health, thereby promoting self-repair. The most common modes of administration are injectable and oral administration. Injectable drug delivery includes intravenous injection, arterial injection, intramuscular injection, and subcutaneous injection; although it has the advantages of being applied to a large number of agents, high bioavailability, and low toxicity, there are shortcomings such as the necessity of frequent administration, difficulties in administering the drugs, and large-dose administration easily causing adverse reactions. Oral drug delivery includes tablets, capsules, and granules, which have the advantages of ease of use, high patient compliance, and easy storage and transportation, but also have the disadvantages of low utilization, slow absorption rates, and the concentration of drugs reaching the target area being low, resulting in poor treatment effectiveness [1,2,3,4,5]. Compared with this systematic system of systemic administration, topical administration shows huge advantages. Topical therapy has the advantages of high local drug concentration, low systemic blood drug concentration, few adverse reactions, and few toxic side effects [6,7]. The most ideal mode of administration is to modify the drug to attain a controlled release function of the drug delivery mode, that is, the drug delivery system (DDS).

The DDS is a technical system that comprehensively regulates the distribution of drugs in organisms in terms of space, time, and dosage, and its main goal is to deliver the appropriate amount of drugs at the appropriate time according to medical conditions so as to maximize the effectiveness of the drugs, improve their efficacy, reduce costs, and reduce toxic side effects [8,9]. The research objects include the drug itself, the carrier materials and devices carrying the drug, and the related technologies for physicochemical modification and modification of drugs or carriers [10]. Modified drug release (modulation release) is compared to regular release, i.e., the rate or timing of drug release, including sustained release, delayed release, controlled release, targeted release, and biphasic release [11,12,13]. Constant release refers to the release of the drug without deliberate modification. Sustained release means that the absorption rate of drugs entering the body is reduced, and the release time is relatively long, which is a non-constant rate of release [14]. Late release means that the drug is not released immediately after administration. Biphasic release refers to the phased release of the drug, the rapid release of the drug in the early stage, and the slow release of the drug in the later stage. Targeted release refers to the targeted delivery of active ingredients to the identified sites for precise drug release, which can control the time, place, and rate of drug release [15].

With the discovery and development of nanotechnology, nano DDSs have attracted increasing attention because of their huge potential. The overall goal of drug delivery using nanocarriers is to effectively treat the disease with minimal side effects, controlling drug release through passive or active targeting in a time-dependent manner at a specific location level [16,17]. At present, electrohydrodynamic jet printing technology, electrospraying technology, and electrospinning technology are all advanced technologies for the preparation of nano-sized to micron-sized materials, which have received widespread attention in the biomedical field [18,19,20,21,22]. Among these, electrospinning technology is the most popular technology. Electrospinning technology is well suited to preparing polymer fibers from the nano to the micron range [23]. It is a technology that uses natural and synthetic polymers to produce nanofiber membranes with diameters ranging from 2 nm to several micrometers [24].

## 2. Electrospinning Technology

The basic principle of electrospinning is to spray the electrospinnable liquid into a strong electric field to form a continuous fiber that involves an electrohydrodynamic process. The basic device of electrospinning consists of four parts [25]: (a) a high-voltage source, (b) a syringe pump, (c) a spinneret, and (d) a collector. In solution electrospinning, in the case of a strong electric field, first due to the surface tension of the solution, droplets are formed on the nozzle of a spinneret, and the surface of the droplets carries an inductive charge [26]. When the electrostatic force is the same as the surface tension of the solution, the shape of the droplet changes from a hemispherical shape to a cone, which is called a Taylor cone [27]. When the electrostatic force is larger than the surface tension, the solution overcomes the surface tension and forms a jet [28,29]. The jet initially extends in a straight line and then undergoes a violent whiplash movement due to bending instability. During the process of reaching the collection unit, only solid fibers are left due to jet stretching and solvent evaporation [30,31,32].

**Figure 1:**
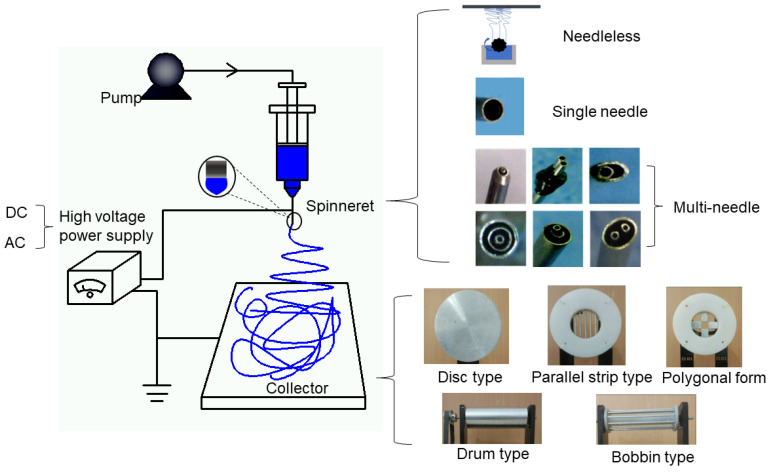
Basic equipment diagram of electrospinning and the influencing factors of process parameters.

In general, the electrospinning process can be divided into four consecutive steps: (i) charging of droplets and formation of a Taylor-conical jet, (ii) a charged jet extending in a straight line, (iii) thinning of the jet and growth of electrical bending instability (also known as whipping instability) in the presence of an electric field, and (iv) solidification and collection of the jet as a solid fiber on a ground collector [33,34]. According to the principle of electrospinning, the electrospinning process can be adjusted by system parameters, process parameters, and environmental parameters to change the morphology and size of the nanofibers [35,36,37]. The system parameters include the type of polymer in the spinning fluid, molecular weight, viscosity, solution conductivity, and surface tension. The process parameters include power supply type (direct current (DC) or alternating current (AC)), receiving distance, collector type [38,39,40], voltage, spinneret type, and flow rate. The environmental parameters include humidity and temperature. Figure 1 shows the basic equipment diagram of electrospinning and the influencing factors of its process parameters [41].

Electrospinning technology can be exploited to treat a wide variety of polymer-based working fluids, including polymeric solutions, emulsions, suspensions, and also polymeric melts [42,43]. Different with a homogeneous solution, an emulsion is a heterogeneous fluid containing two or more immiscible solutions. One exists in the form of a continuous phase, and the other as tiny or ultramicro-sized droplets distributed throughout the continuous phase. Suspensions are mixtures of liquids and suspended solid particles [44]. The treated solutions, emulsions and suspensions can be solidified into fibers through solvent evaporation during the electrospinning processes. However, the polymer melt during a melt electrospinning is non-conductive, and the viscosity is much greater than that of the solution. Unlike the solution electrospinning jet, in-evaporative solidification plays its role and the melt jet is cured by cooling [45,46].

Electrospinning technology can be divided into the following types: single-fluid electrospinning, bi-fluid electrospinning, and multi-fluid electrospinning according to the simultaneously treated number of different kinds of working fluids [47]. Among them, most blended electrospinning and emulsion electrospinning belong to single-fluid electrospinning. Coaxial electrospinning and side-by-side electrospinning are typical bi-fluid electrospinning. Multiple-layer coaxial electrospinnings are often referred to the tri-fluid coaxial electrospinning and four-fluid coaxial process [48,49].

Electrospinning technology can also be divided into needleless electrospinning, single-needle electrospinning, and multi-needle electrospinning according to the different spinnerets. Among these, coaxial electrospinning, parallel electrospinning, and triaxial electrospinning are multi-needle electrostatic spinning modes [50].

**Figure 2:**
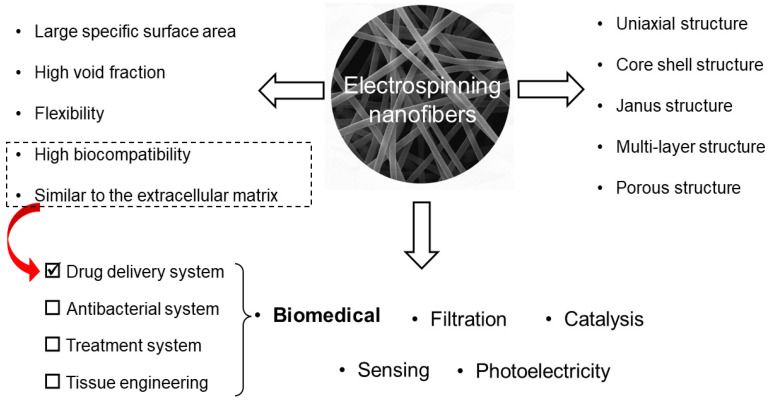
Applications of electrospinning.

Electrospun fibers have different types of structures, such as a uniaxial structure, a core shell structure, a Janus structure, a multi-layer structure, or a porous structure. They have the advantages of a large specific surface area and porosity naturally. Meanwhile, the surface functionalization and performance can be facilely tailored [24,51,52,53,54], thus, it is no strange that electrospinning has been widely explored in the fields of biomedicine [55,56], filtration [57,58,59], catalysis [60,61], energy [62], sensing [63,64], and optoelectronics [65], and particularly in the biomedical field [66,67]. The frequently reported medical devices include DDS [68,69,70], antimicrobial systems [71,72,73,74], therapeutic systems [75], and tissue engineering [76,77]. Additionally, because of their typical high biocompatibility and the fact that their morphology resembles the extracellular matrix of the human body [24,51,52,53,78,79,80], this means that they may be well suited for use in wound dressings [81,82,83,84], tissue engineering [85], and, especially, in vivo administration [86]. Most recently, Ding et al. reviewed the series of interesting applications of electrospun fibrous architectures for drug delivery, tissue engineering cancer therapy and pointed out that many electrospun medical fibers are approaching their real clinic applications as commercial products [86]. For drug delivery applications, electrospun nanofibers are typical intermediate dosage forms [87,88,89,90]. Electrospinning technology has almost no limitations in the selection of polymeric matrices and the encapsulated active pharmaceutical ingredients [91,92,93]. The reported drugs include but not limited to water-insoluble, water-soluble, anticancer, and antibacterial drugs; growth factors; and genetic material [94,95,96,97,98,99,100]. In addition, multi-drug-loaded electrospinning nanofiber membranes can also reduce the number of dosing times, thereby improving patient compliance [101,102,103,104]. Figure 2 shows the advantages, structure, and applications of electrospun fibers.

## 3. Preparation of Drug Nanofibers

Due to the certain particularity of nanofibers in drug transportation, most research in the field of drug carriers gives preference to nanofibers. The most important thing in the preparation of drug-carrying nanofibers is the choice of drug carriers. At present, the carrier materials used for drug delivery in the biomedical field include lipid materials, polymers, inorganic materials, nanoemulsions, and nanocrystals [17,105,106,107,108,109,110,111]. Table 1 shows the size of the drug carrier and its advantages. Among all the nanoscale materials, nanofibers produced from biodegradable and biocompatible polymers have received widespread attention for their flexibility, effectiveness, and unique physicochemical properties [87]. Other popular nanocarriers are mainly nanoparticles and liposomes. Among them, solid lipid nanoparticles are further divided into seven types: monoglycerides, diglycerides, triglycerides, free fatty acids, free fatty alcohols, waxy, and steroids [112].

Currently, a large amount of polymers are successfully being used in electrospinning for the preparation of drug carriers [129,130,131,132]. Polymers can be divided into natural polymers and synthetic polymers according to the source, among which natural polymers are mainly derived from animals and plants [133,134]. According to the different characteristics of the polymers, they can be converted into nanofibers for providing controlled release, sustained release, delayed release, and other kinds of modified release profiles. Table 2 shows the different types of polymers.

**Figure 3:**
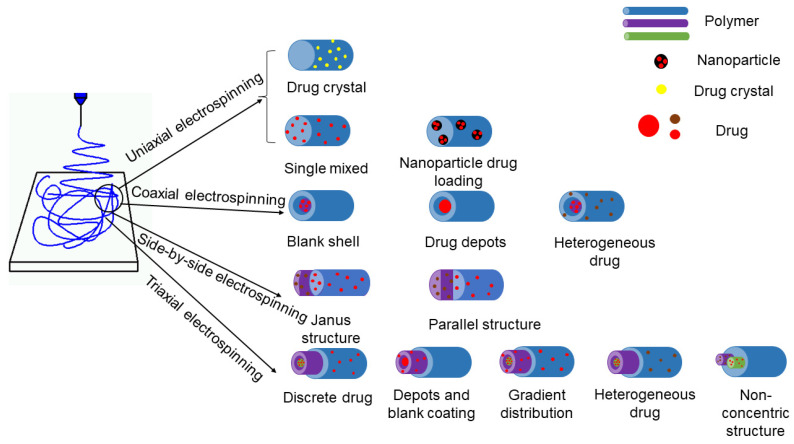
The drug is in the encapsulated form of nanofibers.

Within the nanofibers, a drug can co-exist with a polymer in several manners and in different physical state [135,136,137,138,139]. These cases mainly include: (a) the drug is homogeneously distributed all over the polymeric nanofibers, and most frequently in an amorphous state; (b) the drug distributes on the surface of polymeric nanofibers in a crystal format; and (c) the drug can be firstly encapsulated into the nanoparticles, and then the nanoparticles are loaded into the fiber. They are shown in Figure 3.

## 4. Drug Carrier Technology and Its Controlled Release

In the literature, several drug controlled release mechanisms have been reported, which are manly based on the properties of the drug carriers. These mechanisms include a diffusion mechanism, a swelling mechanism, an erosion mechanism, an erosion mechanism combined with a diffusion and swelling mechanism, an osmotic pressure mechanism, and an ion exchange mechanism. Among them, Fickian diffusion belongs to simple diffusion; swelling control is a physical trigger, caused by factors such as temperature and light, causing the fibers loaded with the drug to swell or degrade, thereby releasing the drug; dissolution control is a chemical trigger, which is highlighted by factors such as degradation and hydrolysis, resulting in the surface erosion of the drug-carrying fibers to achieve drug controlled release [140]. The osmotic pressure mechanism is the use of osmotic pressure so that the drug evenly penetrates outward. The ion exchange mechanism operates by water-insoluble cross-linked polymer resin; when the drug-carrying resin containing the appropriate charge ions comes into contact with the solution, the drug molecules are exchanged and diffused into the solution. The most important drug release mechanisms are the diffusion and erosion mechanisms. Three other major mechanism for controlling drug release are the following: networking, matrix embedding, and coating [9]. Different drug-carrier fibers prepared by different drug-loading techniques have different release mechanisms. In the following, controlled drug release and its controlling factors are discussed in detail in relation to electrospinning using a number of fluids.

### 4.1. Uniaxial Electrospinning Drug Carrier

#### 4.1.1. Solution Electrospinning

Solution electrospinning requires a solvent or a solvent mixture, in which both the filament-forming polymer and the drug can reach a certain concentration for an electrospinnable property of the solution and the therapeutic effect of the drug after administration [141]. In addition, because various types of materials and drugs can be processed by electrospinning in solutions, they are not limited to the use of polymers and drugs and require only simple equipment. Solution electrospinning is the most researched and applied technology for loading drugs in electrospinning fibers [142]. Solutions in electrospinning are usually mixed solutions.

Blend-solution electrospinning was developed on the basis of traditional electrospinning; a variety of drugs can be encapsulated into electrospinning nanofiber membranes, and it is the simplest method, belonging to uniaxial electrospinning. According to relevant reports, the electrospinning process has no effect on the biological activity of antibiotics [143] and polyphenols [144]. However, due to high permeability and shear stress, the structure of the liposomes is destroyed during the electrospinning of the mixed solution [145]. In general, there is an initial explosion phenomenon in the process of the electrospinning of blends, which is mainly due to the distribution of the drugs on the surface of the fibers during the electrospinning process, and the surface volume of the electrospinning nanofiber membrane is relatively high [146].

**Figure 4:**
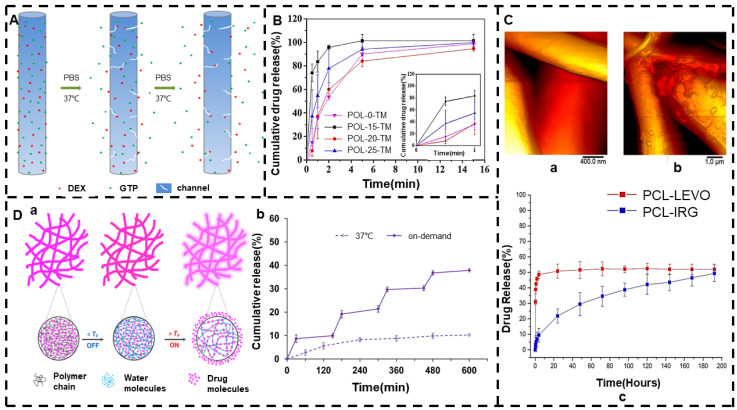
Solution electrospinning. (**A**) Release mechanism of GTP and DEX wrapped in phosphate-buffered aqueous solution at 37 °C (reprinted with permission from [150]. Copyright©2014, Springer Science Business Media New York). (**B**) At 35 °C (n = 3; AFM images of PCL-IRG fibers and PCL-LEVO fibers at STF pH 7.4 at mean ± SD) (reprinted with permission from [151]. Copyright©2022, American Chemical Society). (**C**) (**a**–**c**) PCL-IRG fibers and PCL-LEVO fibers at STF pH 7.4; percentage of cumulative drug release released by IRG and LEVO in phosphate-buffered saline media (reprinted with permission from [152]. Copyright©2019 Elsevier B.V. All rights reserved). (**D**) (**a**) Release mechanism of thermally triggered electrospinning membranes with switching function. (**b**) In vitro pulsating release curve of RhB of F3 composite nanofibers during heating (45 °C)/off (37 °C) cycle while taking the release curve at 37 °C for comparison (reprinted with permission from [149]. Copyright©2019 Elsevier B.V. All rights reserved.).

Electrospinning mixed solutions can load drugs with different properties into the same membrane, and a nanofiber membrane with biphasic drug release is prepared by the properties of the drug itself to achieve controlled release of the drug. Biphasic drug release is manifested by rapid release first and sustained release later, reducing the time required to administer the drug [147,148]. The initial dissolution of the drug is due to the appearance of the drug on the surface in an amorphous form, determined by the solubilizing effect and leaching action. Stable releases are due to longer diffusion paths [149]. In the solvent system of acetone and DMF, Li et al. [150] mixed the hydrophilic drug GTP and the hydrophobic drug DEX with PLGA to prepare an electrospun nanofiber membrane with biphasic release. This experiment confirmed that DEX molecules can successfully be released from channels formed by early-release GTP molecules. The release mechanism is shown in Figure 4A, and the two mechanisms involved in GTP and DEX release are the diffusion of drug molecules and the degradation of polymers. The study used the pro-hydrophobicity of the drug to achieve biphasic release of the drug.

Solution electrospinning can also be used to prepare nano drug-loaded fiber membranes with a local drug delivery function, which can control drug release due to the properties of the polymer. Topical administration is an accurate means of administration that can better reduce the toxic side effects to the whole body and also has a more convenient frequency of administration, which is conducive to the sustained release of drugs and improving patient compliance. Andreadis et al. [151] prepared an in situ gelatinous nanofiber film containing PVA and Poloxamer 407 by electrospinning for ocular delivery of TM. This experiment studied the in vitro release curve of TM from electrospun nanofibers, as shown in Figure 4B. Due to the hydrophilic nature of PVA nanofibers, the fibers belong to Fickian diffusion control, allowing the drug to be released quickly. To this end, the controlled release of the drug was achieved due to the hydrophilic and hydrophobic properties of the polymer.

Different drugs can change the internal structure of polymeric substrates by solution electrospinning. The electrospun nanofiber membrane prepared by the same solution will have different drug release effects by loading different drugs and using different encapsulation forms. Hall Barrientos et al. [152] used chloroform and DMF as solvents to prepare PCL electrospun fiber membranes loaded with irgasan and levofloxacin by electrospinning. In vitro drug release studies have shown that PCL-IRG fiber-released irgasan exhibits sustained release behavior, indicating that the drug is released through molecular diffusion, possibly due to the hydrophobicity of the drug, so its membrane also has some degree of hydrophobicity. The release of levofloxacin by PCL-LEVO fibers exhibited explosive release behavior, possibly due to the hydrophilicity of levofloxacin and the way the drug adsorbs on the surface of the polymer. The AFM image and release curve are shown in Figure 4C.

Blended electrospinning can also be used to produce other novel “smart” drug-loaded nanofiber membranes that vary in temperature, allowing pulsatile drug release on demand. Amarjargal et al. [149] used RhB as a drug model, added different proportions of PMMA and ERS to the blend polymer, changed its glass transition temperature (Tg), and successfully prepared a new type of easily processed heat-sensitive ERS/PMMA membrane. Thus, the best drug release effect can be obtained under physiological temperature- and heat-triggered drug release conditions. The drug release mechanism of the thermally triggered electrospinning membrane with a switching function is shown in Figure 4D(a). By studying the pulsating drug release behavior of composite nanofiber F3 under the action of a reversible glass–rubber transition mechanism, the on/off switching release of this drug was preserved in four repeated temperature-driven cycles, confirming the realization of on-demand pulsation release; the in vitro release curve is shown in Figure 4D(b). All the above-mentioned electrospinning processes were conducted by treating only a single solution as the working fluid. The chemical and physical properties of filament-forming polymeric matrices were exploited to modify the loaded drugs’ release behaviors.

#### 4.1.2. Emulsion Electrospinning

**Figure 5:**
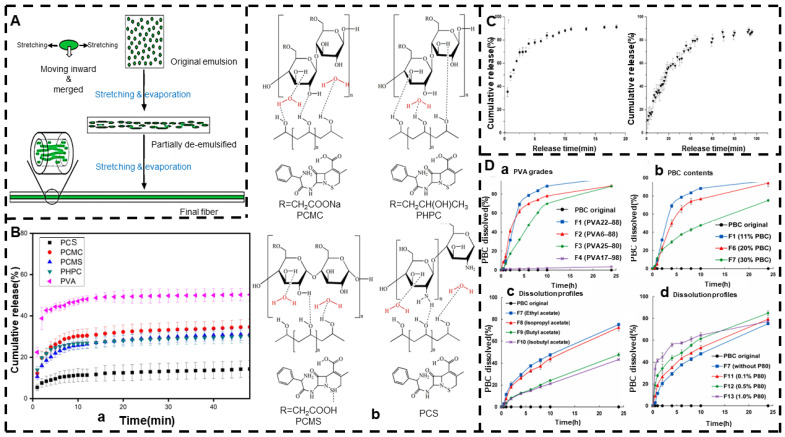
Emulsion electrospinning. (**A**) Schematic diagram of the mechanism of core–shell fiber formation during emulsion electrospinning (reprinted with permission from [158]. Copyright©2006, WILEY-VCH Verlag GmbH & Co., KGaA, Weinheim). (**B**) (**a**) In vitro drug release of PVA/biopolymer mixtures and (**b**) intermolecular interactions between PVA and different biopolymers (reprinted with permission from [164]. Rights managed by Taylor & Francis). (**C**) In vitro drug release curve of PCL fibers containing KET and PCL/gelatin crosslinked fibers (reprinted with permission from [166]. Copyright©2017, Elsevier B.V. All rights reserved.). (**D**) (**a**–**d**) Release curves of PVA grade, PBC content, use of different solvents, effect of P80 concentration on PBC dissolution of PPA nanofibers (reprinted with permission from [170]. Copyright©2021, Elsevier B.V. All rights reserved).

Emulsion electrospinning is a uniaxial electrospinning technique, but nanofibers with a core–shell structure can be prepared [153]. The electrospun nanofiber membrane prepared by emulsion electrospinning is released faster in the shell than in the core layer. It plays a role in protecting bioactive substances and allows the sustained release of multiple drugs [154,155]. When two drugs are simultaneously located in the core layer of nanofibers, the release of one drug is affected by the release of another drug [156]. Emulsion electrospinning fibers are less toxic and contribute to the adhesion and proliferation of fibroblasts [157]. In the electrospinning process for emulsions, the drug is usually dissolved in the aqueous phase and then dispersed in an organic polymer solution containing a suitable surfactant, i.e., the oil phase, because the evaporation rate of the oil phase is faster than that of the aqueous phase, resulting in a higher viscosity of the oil phase, and then the aqueous phase moves inside, while the oil phase moves to the edge of the nanofibers due to the viscosity gradient, i.e., stretching and evaporation-induced demulsification occurs, nanofibers with a core–shell structure are prepared, and most of the drugs are encapsulated in the fibers [158,159]. In the emulsification process, hydrophilic drugs are dissolved in water (aqueous phase), while hydrophobic polymers are dissolved in solvents (oil phase), so this process can effectively alleviate or even avoid the phenomenon of drug explosion. In addition, the process can produce good-quality nanofibers from diluted polymer solutions [160,161]. A schematic diagram of the mechanism of the formation of nanofibers in the core–shell structure using the electrospinning process for emulsions is shown in Figure 5A.

Emulsion electrospinning provides a green method that can be used to prepare drugs using simple equipment to control the release of nucleus–shell nanofibers, which is a kind of “green electrospinning”. The so-called “green electrospinning” process involves electrospinning polymers in an aqueous solution to avoid harmful organic solvents [162,163]. Since the interactions between polymer chains affect drug release to a certain extent, different polymers exhibit different controlled-release effects. Hameed et al. [164] blended PVA with CS, CMC, CMS, HPC, and other biopolymers, respectively, and prepared cephalexin-loaded nanofibers with a core–shell structure using emulsion electrospinning. Due to the differences in the interactions between the PVA chains, the release of cephalexin was in the following order: PCS < PCMS < PHPC < PCMC, and they were all controlled by Fickian diffusion, as shown in Figure 5B.

Solution electrospinning has some defects in the burst release of loaded drugs. Emulsion electrospinning can be used to prepare electrospun fibers with continuous and sustained release functions to alleviate the burst release of drugs. By adding emulsion droplets, various lipophilic components can be easily loaded into the hydrophilic polymer matrix of the fibers to avoid bursting phenomena [165]. Basar et al. [166] prepared KET-loaded PCL and PCL/gelatin electrospun fibers using solution electrospinning and emulsion electrospinning. After crosslinking, the drug release property was compared. In vitro drug release studies showed that binary PCL/gelatin electrospun fibers completely inhibited the burst release of KET and exhibited continuous and sustained drug release for up to 4 days, as shown in Figure 5C. This is due to the fact that the drug release from the PCL fibers belongs to Fickian diffusion, while the drug release from the PCL/gelatin fibers belongs to non-Fickian diffusion. Therefore, the drug controlled-release effect can be achieved by adding polymers. The role of gelatin, for example, is to provide a function through cross-linking and then act as an effective barrier against the diffusion of drugs. Similarly, Koosha et al. [167] showed that the sustained release of drugs can be effectively achieved through crosslinking.

Emulsions can be converted into a nanofiber-based solid dispersion through electrospinning. The solid dispersion is a dispersed mixture of one or more APIs in a solid inert carrier [105,168]. Solid dispersions are a versatile strategy that can be used to develop oral solid dosage forms of poorly soluble drugs and also to increase the rate of in vitro dissolution of poorly water-soluble drugs [169]. Shibata et al. [170] used an oil/water (O/W) emulsion consisting of PBC dissolved in ethyl acetate and PVP aqueous solution for electrospinning to improve the solubility of the PBC. In this study, the surface PVA grade, PBC content, O/W emulsifying solvent, and added surfactant content were found to affect solubility and other properties, and the release curve is shown in Figure 5D. The fiber was controlled release by swelling, and the drug was encapsulated in the fiber in an amorphous form, which improved the solubility of the drug to a certain extent. Gelb et al. [171] also investigated the effects of polymer type, polymer molecular weight, solution concentration, and incorporation of the model drug Cip HCl on the delivery properties of electrospun nanofibers. Therefore, the controlled-release effect and drug dissolution enhancement can be achieved through polymer properties (such as degree of polymerization and degree of hydrolysis), drug content, and by adding different concentrations of surfactant.

#### 4.1.3. Melt Electrospinning

Melt electrospinning is a solvent-free method that involves the direct mixing of drugs in polymer melts [172]. However, most biomolecules such as proteins, polysaccharides, nucleic acids, and thermal polymers cannot be electrospun by melt electrospinning. Fibers prepared from melt electrospinning are typically micron sized or larger in diameter and require no downstream processes such as screening [173,174]. This process has the advantages of no solvent residue or release, good biomedical safety, and a large drug load [142]. In general, during the uniaxial elongation of the molten polymer, the jet is unstable with a decrease in the viscosity of the polymer, so the heating time during the drawing process should be shorter because when the heating time is longer, the polymer temperature is higher, that is, the polymer viscosity is lower, and the long-term drawing makes the jet unstable [175].

Unlike solution electrospinning devices, melt spinners require more complex equipment, often with higher rotational voltages and heating systems including temperature sensors. In the melt electrospinning process, one electrode is connected to the spinning melt and the other electrode is connected to a metal collector. When electrostatic power overcomes the surface tension of the polymer melt, the discharge jet erupts and elongates in the electrostatic field, where it then cools and is deposited on the collector [176].

**Figure 6:**
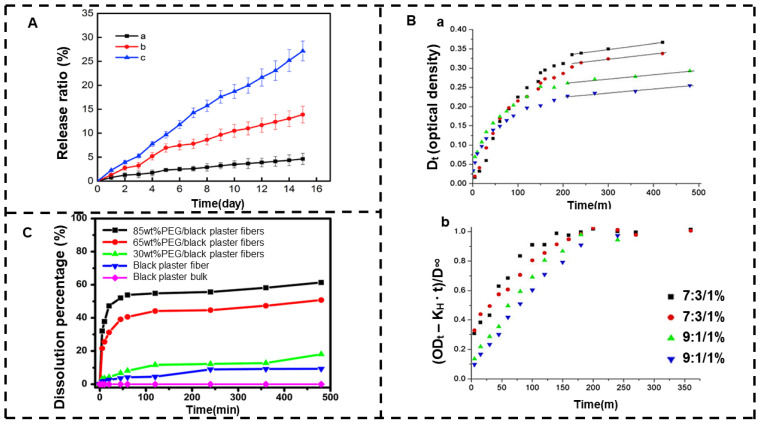
Melt electrospinning. (**A**) Drug release curve of PCL melt electrospun fibers containing daunorubicin hydrochloride: (a–c) 1wt%, 5wt%, and 10 wt% (reprinted with permission from [177]. Copyright©2017, the authors. Production and hosting by Elsevier B.V. on behalf of KeAi Communications Co., Ltd.). (**B**) (**a**) Kinetic characteristics of drug release of PLLA/PHB pads and (**b**) effect of diffusion on controlled drug release (reprinted with permission from [178]. Copyright©2017, Controlled Release Society). (**C**) Dissolution rate of Draconis Sanguis in vitro (reprinted with permission from [179]. Copyright©2019, Wiley Periodicals, Inc.).

The drug loading of electrospun nanofibers is often limited by drug solubility and solvent concentration in mixed-solvent systems, while melt electrospinning is a solvent-free method, so new drug delivery systems can be developed through the melt electrospinning method to solve the solvent limitation problem. Melt electrospinning can use its filamentation principle to cause polymer molecular chains to rearrange themselves and thus carry out the controlled release of drugs. Lian et al. [177] dispersed the hydrophilic drug daunorubicin hydrochloride in a PCL fused electrospun fiber membrane in a clustered state. In the process of melt electrospinning, the PCL molecular chain is rearranged to form crystals, which inhibit the penetration of water and the diffusion of drugs due to the high crystallization and hydrophobicity of its fiber membrane, thus avoiding the sudden release of drugs. After a period of time, the molecular chains are loosely arranged, and the water is able to diffuse within the fibers through amorphous areas, releasing a large amount of the drug. For this reason, the fiber has a slow release rate and a long-term release period. The release curve is shown in Figure 6A.

Melt electrospinning can carry the drug into polymer fibers with biodegradability, and the drug is controlled by hydrolysis reactions. Gao et al. [178] prepared PLLA/PHB fibers with different concentrations of the DPD drug using fused electrospinning. Figure 6B shows the release curve. During hydrolysis, encapsulated drugs enter the aqueous environment through diffusion mechanisms and surface degradation. The 9:1 PLLA/PHB system was more resistant to polymer hydrolysis than the 7:3 system. The diffusion transfer rate of 7:3 PLLA/PHB fibers was about two times higher than that of the 9:1 system, which was related to its crystallinity. Therefore, the release of the drug can be controlled by the content component ratio of the two polymers.

Melt electrospinning can load poorly water-soluble drugs onto the fibers, thereby increasing the dissolution rate of the drug and the delivery rate of the drug. The higher the viscosity of the melt, the greater the adhesion of the produced fibers and the greater the diameter. However, when the fiber diameter is too large, it is not conducive to the dissolution of drugs with poor water solubility. Controlled release of the drug can also be performed by obtaining the diameter of the fiber according to the viscosity of the melt. Xu et al. [179] prepared different proportions of ultrafine black gypsum fibers and black gypsum/PEG composite fibers using a novel upward melting electrospinning technique. The in vitro drug dissolution results showed that the solubility of Draconis Sanguis, which has poor water solubility, was significantly improved, and the structure of the microfibers and the incorporation of PEG promoted the in vitro release of insoluble drugs in black plaster. The drug release curve is shown in Figure 6C. The mechanism involved in the release of this fiber drug is the degradation of polymers and the diffusion of drug molecules. Therefore, the controlled-release effect of the drug was achieved by changing the polymer concentration.

### 4.2. Coaxial Electrospinning Drug Carrier

In simple electrospun fiber membranes, the distribution of drugs on the surface of nanofibers, the large surface area of the nanofibers, and the amorphous state of the drugs in the nanofibers all lead to an initial explosive release, which is not ideal for the continuous release of drugs [180]. In order to eliminate the explosive release, post-processing such as cross-linking or chemical modifications of nanofiber membranes is needed. However, these types of post-treatments may lead to a decrease in toxicity and biocompatibility [181]. Coaxial electrospinning is another method by which to eliminate explosive release that is superior to post-processing [182]. Core–shell nanofibers can be formed by the simultaneous delivery of two different fluids through coaxial electrospinning, where one polymer is coated by another polymer, thereby improving the performance of both polymers at the same time [50,183,184]. Hollow fibers with different internal and external components and functional fibers with possible coatings can also be obtained [185,186]. In addition, a small amount of non-spinnable material can be electrospun into core–shell fibers [187].

**Figure 7:**
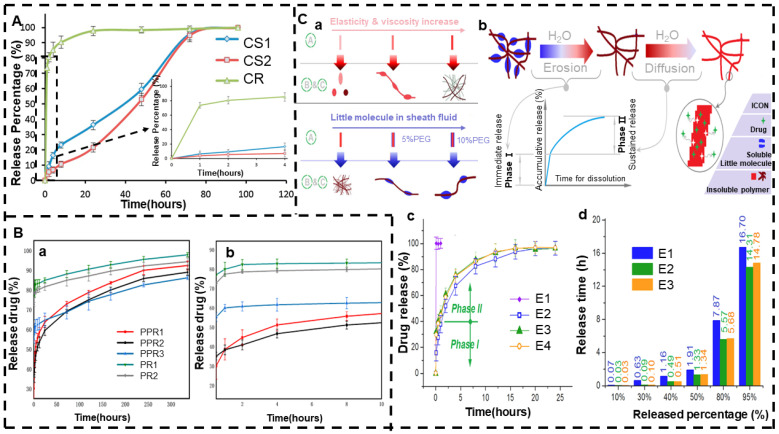
Coaxial electrospinning. (**A**) CS, CS1, and CS2 fiber membrane in vitro drug release curve (full range and first 4 h) (reprinted with permission from [188]. Copyright©2016, Elsevier B.V. All rights reserved). (**B**) ROX drug release curve: (**a**) 14 days and (**b**) 10 h (reprinted with permission from [192]). (**C**) (**a**) Formation mechanism of engineering SOS, (**b**) overview of biphasic release of typical drugs, (**c**) percentage of drug release curve vs. time, (**d**) time required to release a certain amount of the loaded drug from an EHDA product (reprinted with permission from [194]. Copyright©2021, Donghua University, Shanghai, China).

Coaxial electrospinning has a controlled-release effect to a certain extent due to the nanofibers produced by the nucleus–shell structure. Its use in controlled drug release can also be extended to polymers and drugs with low compatibility. Controlled release is achieved by changing the thickness of the shell because the shell acts as a barrier to diffusion. Zahida et al. [188] prepared PCL nanofibers loaded with the hydrophilic drug ampicillin with shell fluid that could not be fully electrospun and core liquid that was fully electrospinnable. By changing the flow rate of the shell liquid, it was found that samples with a larger shell flow rate showed a slower initial release, possibly due to the thicker fibrous shell layer produced by the larger flow rate. PCL is non-expandable and has a very slow degradation rate within PBS, so the fiber has a Fickian diffusion mechanism, and its release curve is shown in Figure 7A. In addition, this process has the advantage of smaller fiber diameters and less clogging of the needle tip. Therefore, the fiber thickness can be adjusted by changing the housing flow rate, which can be exploited to modify the the drug release profiles. Liu et al. [189] also confirmed this case using a modified tri-axial electrospinning.

As previously mentioned, coaxial electrospinning can alleviate the explosive release of drugs, but coaxial porous nanofibers and uniaxial electrospun nanofibers also exhibit certain phenomena of the explosive release of drugs. Coaxial porous nanofibers can effectively alleviate the problem of drugs’ explosive release and achieve a sustained release of the drugs [190]. The drug release performance of porous microfibers was found to be better than that of non-porous microfibers [8,191], and the release of the drugs was regulated by porosity, so the porous structure increased the dissolution of drugs. Chen et al. [192] prepared ROX-loaded PCL/PLA porous core–shell nanofibers using coaxial electrospinning technology and non-solvent-induced phase separation and compared them with porous core–shell nanofibers and uniaxial porous nanofibers. The results showed that the porous core–shell nanofibers not only slowed down the burst of the drug, but also increased the dissolution of hydrophobic drugs, as shown in the release curve in Figure 7B.

Coaxial electrospinning can also be used to achieve drug-loaded nanofiber membranes with a duplex drug release function [193]. Unlike the previously mentioned solution electrospinning that uses the pro-hydrophobicity of the drug to achieve biphasic release, coaxial electrospinning is achieved using its special structure. He et al. [194] used an improved coaxial electrospinning method to prepare a hybrid material with a PEG small-molecule solution as the sheath solution and an EC solution as the core liquid, and both contained an IBU nanofiber structure hybrid material, that is, engineering spindles-on-a-string (SOS) products. SOS products have a typical biphasic drug release curve, and their formation principle and biphasic release profile of typical drugs are shown in Figure 7C; additionally, the main mechanism of drug release is the Fickian diffusion mechanism. An in vitro dissolution test confirmed that SOS products can provide a typical two-stage release mode; the first stage is due to the solubility of PEG, immediately releasing 40% of the load of IBU within 1 hour, and, in the second stage, the drug is continuously released by the remaining loads encapsulated in the insoluble EC.

### 4.3. Side-by-Side Electrospinning Drug Carrier

Janus nanofibers can be prepared using side-by-side electrospinning. They can be prepared by electrospinning using side-by-side needles, but this method is not easy to carry out. Because the two working fluids in side-by-side electrospinning are simultaneously located under an electric field with the same negative or positive charge, this makes it easy for them to repel and separate from each other [195]. Side-by-side electrospinning has traditionally been performed under conditions where the bifurcate has a certain viscosity and conductivity [196,197,198].

Side-by-side electrospinning is a useful tool to combine different polymeric matrices for providing biphasic drug release. For example, the drug can be encapsulated into both sides of the fibers with one side of a water-soluble polymer and the other side of a water-insoluble polymer. The side of soluble polymer can release the drug in an erosion mechanism, and the side of insoluble polymer can manipulate the drug release behavior in an extended manner, and thereby a typical biphasic release can be ensured. Along this way, many novel combinations can be conceived for furnishing multiple-phase release profiles of one drug.

**Figure 8:**
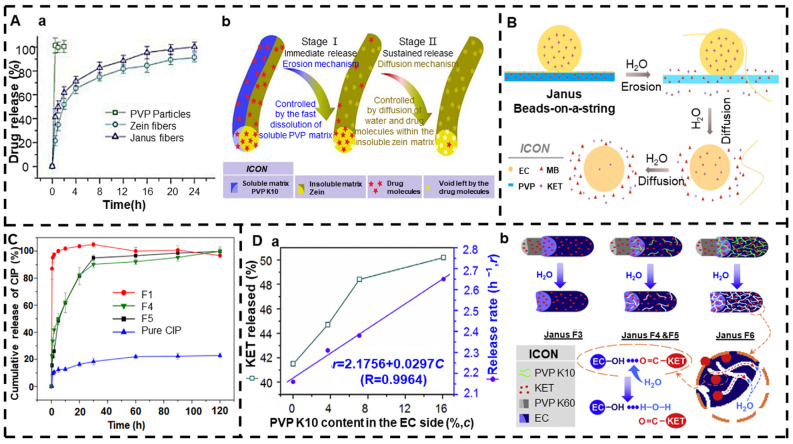
Side-by-side electrospinning. (**A**) (**a**) In vitro drug release curve and (**b**) mechanism by which Janus fibers provide a biphasic release curve (reprinted with permission from [199]. Copyright©2020, Elsevier). (**B**) Drug release mechanism diagram (reprinted with permission from [200]). (**C**) In vitro release curve of CIP from drug−-loaded fibers and pure drug granules (reprinted with permission from [201]. Copyright©2020, Elsevier B.V. All rights reserved.). (**D**) (**a**) Phase II drug release curve and (**b**) Janus fiber release drug mechanism (reprinted with permission from [202]. Copyright©2015, Elsevier B.V. All rights reserved.).

Wang et al. [199] prepared a biphasic advanced drug delivery fiber that released FA by loading the drug FA on both sides of the Janus nanofiber formed by the combination of food polymer corn protein and PVP using side-by-side electrospinning. The drug release curve and biphasic drug release mechanism are shown in Figure 8A. The first stage is the rapid release of FA from the PVP side through the erosion mechanism. The amount of release from the PVP side can be adjusted by adjusting the FA concentration in the working fluid prior to manufacturing. The second stage is the continuous release of FA from the zein side through the diffusion mechanism. Since zein is an insoluble macromolecule, the water molecule penetrates the skeleton of the zeatin, causing the FA molecule to be released from the hydrogen bond with the zeatin molecular group. Then, the free FA molecules penetrate outward into the bulk solution in the fibers.

Li et al. [200] used side-by-side electrospinning to combine PVP K90 with EC to produce Janus bead string products using KET and MB as model drugs. In vitro dissolution tests showed that Janus beaded products also offer a typical dual-drug controlled-release curve as compared to Janus nanofibers, providing the immediate release of MB and sustained release of KET. Figure 8B is a schematic diagram of the drug release mechanism. As with the drug release mechanism described above, the hydrophilic side is a typical erosion mechanism that releases all drugs. The hydrophobic side provides a sustained release of the drug for a typical diffusion mechanism. Additionally, the study also demonstrated that by varying the polymer concentration, Janus beads with different particle distributions and diameters can be obtained. Similarly, Yang et al. [201] combined PVP K90 and EC into Janus fibers using side-by-side electrospinning. The difference was that what they prepared was not biphasic release fibers, but fibers with the immediate release of the drugs. Figure 8C shows the in vitro release curve of the Janus fibers co-loaded with CIP and AgNPs.

Yu et al. [202] prepared a nanofiber membrane with a biphasic drug release function and an adjustable release rate in the second stage using Teflon-coated parallel spinnerets. This is the same as the combination of PVP K60 and EC, but the difference is that PVP K10 was added on the EC side to accelerate the release of the second stage. Studies have shown that doped hydrophilic PVP K10 can easily manipulate the rate of drug release of the EC matrix. The drug release curve and fiber release mechanism of the second stage are shown in Figure 8D. Later, Yu et al. [203] developed a structured spinneret for the three-fluid electrospinning process, but still no application reports about the electrospun complicated nanostructures.

### 4.4. Triaxial Electrospinning Drug Carrier

The three-layer coaxial electrospinning (triaxial electrospinning) system mainly includes a high-voltage power supply, a spinneret, a collector, and a fluid drive pump [204,205]. The products prepared by triaxial electrospinning technology are three-layer nanofibers. Triaxial electrospinning uses three concentric needles of the spinneret, and the three fluids driven by the different drive pumps are external liquid, intermediate liquid, and core liquid, which meet at the tip of the spinneret; the working principle of this process is the same as that of other electrohydrodynamic atomization methods such as coaxial electrospinning [206,207]. Triaxial electrospinning technology has been developed in various forms, with core polymers surrounded by two different layers of polymers and core polymers surrounded by two layers of the same polymer; there are also core polymers surrounded by an outer layer and a layer of void areas. Liu et al. [208] prepared biodegradable nanofibers with a multilayer structure of gelatin/PEG/gelatin using triaxial electrospinning technology. Growth factors or drugs are incorporated into the core and are released by diffusion and degradation. The three layers of nanofibers can control drug release by containing different types of drugs in each layer.

Triaxial electrospinning technology is a simple method that enables the preparation of functional nanofibers with complex structural features [209]. The use of triaxial electrospinning can be prepared for the linear release or zero-level release of drug-carrying nanofibers, thereby eliminating the phenomenon of drug burst release; additionally, for a long time after administration, the drug can be maintained within a specific therapeutic concentration so as to improve patient compliance and achieve treatment effects with minimal side effects.

**Figure 9:**
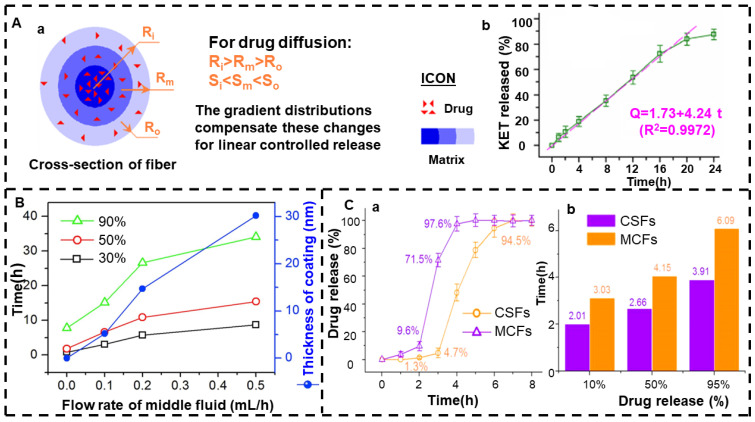
Triaxial electrospinning. (**A**) (**a**) Drug gradient distribution yield linear release curve and (**b**) in vitro dissolution test results (reprinted with permission from [210]. Copyright©2015, American Chemical Society). (**B**) Flow rate, coating thickness, and release time of the intermediate fluid (reprinted with permission from [211]. Copyright©2018, Elsevier B.V. All rights reserved). (**C**) Drug release performance in vitro: (**a**) cumulative aspirin release as a function of drug release time points and (**b**) time required to achieve a certain percentage of aspirin loading in the prepared nanofibers (reprinted with permission from [213]).

Yu et al. [122,210] used triaxial electrospinning to fabricate three layers of nanofibers containing EC in each layer, but the KET concentration content in each layer was different, and it gradually increased from the outer layer to the inner layer, showing a drug gradient distribution. Since EC is insoluble in water, its release mechanism is diffusion release. Figure 9A shows the drug release principle and release curve. As the surface area of the fiber from the nucleus layer to the shell gradually increases (Si < Sm < So), the distance of the drug molecules from the dissolving medium through diffusion also increases gradually (Ri > Rm > Ro). These two factors tend to lead to an initial burst, but with the gradual increase in drug concentration, the effects on the sustained release of the nanofibers can be counteracted, thus achieving the desired release effect. Therefore, controlled release can be achieved by changing the content of the drug.

Liu et al. [211] prepared high-quality three-layer nanofibers using two non-spinnable solutions, i.e., acetone and acetic acid, as the external solution; CA as the intermediate solution; and FA and gliadin as the core solution. The thickness of the CA coating can be precisely adjusted by the flow rate of the intermediate working fluid. Figure 9B shows that the thickness of the CA coating itself is closely related to the flow rate of the intermediate fluid. The CA coating eliminates the initial burst release of the single FA-gliadin fibers and also results in a near-zero-order release curve that can be progressively adjusted by changing the thickness of the coating. In addition, Yang et al. [212] also prepared a three-layer core–shell nanofiber with a CA intermediate solution and FA as a model drug through triaxial electrospinning. The fiber was able to provide near-zero-order release within 36 h without an initial burst release.

In addition to the above, triaxial electrospinning can also be used to prepare drug-loaded nanofiber membranes with targeted drug release functions. Targeted drug release involves targeting the drug to the lesion site, with little impact on non-target organs, tissues, and cells, which not only improves the efficacy but also reduces the toxicity and side effects of the drug. Ding et al. [213] used a new and improved triaxial electrospinning technology to prepare a core–shell nanofiber (CSF) based on ES100, which was loaded with aspirin. It was compared with the monolithic composite nanofiber (MCF) prepared using traditional single-fluid electrospinning. The aspirin release curves of the MCF and CSF nanofibers are shown in Figure 9C. In vitro dissolution assays showed that CSF prolonged the release time of aspirin, protected the first 2 h of gastric membrane under acidic conditions, and prolonged the sustained release time of aspirin in a posterior neutral environment, thereby avoiding excessive cytotoxicity to the circulatory system. Both nanofibers are part of an erosion mechanism that releases drugs. Compared with the double-layer core–shell nanofibers, the tri-layer core–shell nanofibers can support more possibilities for developing novel functional nanoproducts.

### 4.5. Other Drug-Carrier Technologies

In addition to the above preparation methods of electrospun nanofibers, more ideal controlled drug release systems can be achieved using a combination of electrospinning and other fabrication techniques. Table 3 shows these combinations, in which drug release mechanisms and their highlights are explained in detail. Among these combined techniques, physical methods such as electrospraying and 3D printing are popular [214,215,216,217,218,219,220,221,222,223,224].

Liu et al. [224] prepared a drug-loaded double-layer mixed film with biphasic controlled release by combining the hybrid electrospinning and flow film method. The membrane releases the drug in a pulsating manner in the first stage, which belongs to the control of the erosion mechanism. In the second stage, it releases residual drugs in an extended manner through a typical Fickian diffusion mechanism. There are no limitations for these combinations [225]. As an interdisciplinary field, pharmaceutics shows a strong trend in that new excipients and advanced techniques are continuously being drawn upon for the creation of functional medicated materials to modify drug release profiles [226,227]. All the introduced techniques and excipients may have the potential to be combined with electrospinning for the development of brand-new functional nanomaterials. Particularly, some new materials such as hydrogels [228,229,230] and even inorganic nanoparticles may enrich the electrospun nanofiber-based functional biomaterials, and thereby provide novel strategies for resolving the challenges of treating difficult miscellaneous diseases [231,232,233].

## 5. Conclusions and Prospective

In recent decades, with the development of electrospinning technologies such as solution electrospinning, emulsion electrospinning, melt electrospinning, coaxial electrospinning, triaxial electrospinning, and side-by-side electrospinning, electrospun nanofibers have shown great advantages as drug carriers and are widely used in the field of drug delivery systems. By choosing different electrospinning methods, nanofibers of different structures can be prepared, drugs of similar properties can also be loaded, and drugs of different properties can also be loaded at the same time. By combining electrospinning with other preparation techniques, nanofibers with different properties can be prepared. In addition, by combining the properties of the drug itself, the post-treatment process, the polymer properties, the drug-carrying technology, and changes in the process parameters, the release of the drug can be quick, continuous, or continuously adjusted to achieve targeted release, bidirectional release, controlled release, pulse release, sustained release, and other types of drug release; the preparation of nanofibers with linear release or zero-level release functions is also possible.

At present, the research on drug release and drug delivery systems in relation to electrospinning methods almost all concerns the release of drugs in vitro; the release of drugs in a complex in vivo micro-environment is not clear but can be explored in combination with animal experiments. Electrospun nanofibers have good development prospects in amplification technology. Most electrospun nanofibers exist only in laboratories; how to produce electrospun nanofiber products on a large scale, and whether there are good prospects for their development on the industrial scale remain questions to be answered. In addition, degradable electrospun nanofiber materials have relatively few practical clinical applications. How to match the degradation characteristics of electrospun nanofiber membranes with tissue repair remains a huge challenge, so there is great potential in clinical applications. However, with the rapid development of science and technology, all of these problems are expected to be solved in the future.

## Figures and Tables

**Table 1:** Size and advantages of drug carrier materials.

Name	Diameter/Aperture	Advantage	References
Solid lipid nanoparticles	100–1000 nm	Low toxicity, highly effective drug targeting, controlled-release drugs, high drug loads (especially lipophilic drugs), prevents degradation and has versatility	[112,113,114]
Lipidosome	50–200 nm	Biocompatible, biodegradable, non-toxic and non-immunogenic	[115]
Dendritic polymer	1.5–14.5 nm	Spherical homogeneous structure, high biocompatibility, lipophilic, variable composition	[116]
Nanocapsule	10–1000 nm	Improves efficacy and bioavailability, prevents drug degradation, and provides controlled-release delivery	[117]
Polymeric micelle	10–100 nm	Improves bioavailability, alters drug release curves, and improves patient compliance	[118]
Mesoporous silica material	2–50 nm	Good biocompatibility, large specific surface area, large porosity, high drug carrying capacity, good thermal and chemical stability, can carry hydrophilic and lipophilic drugs	[119,120,121]
Carbon nano tube	0.4–2 nm	Water solubility, biocompatibility, low toxicity, high drug load, intrinsic stability, high specific surface area	[122,123,124]
	2–100 nm		
Nano-emulsion	Submicron order	High stability, high load capacity, improved solubility and bioavailability	[125]
Nanocrystal	1–1000 nm	Stabilized by surfactants or stabilizers, no need for carrier materials, drug nanocrystals can enhance the adhesion to biofilms, increases the saturation solubility of drugs, large specific surface area, high bioavailability, high stability, high drug loading capacity, stable dissolution, sustained release drugs and safety	[126,127,128]

**Table 2:** Different types of polymer carriers.

Natural Polymers	Water-Soluble	Water-Insoluble	Degradable	Small Molecule
Botany	Alginate	Cellulose	Glucan	CD
	Zein	NR		
	Pectin			
Animal	Gelatin (soluble in hot water)	Collagen	PASP	
			CS	
Microorganism	PGlu			
Synthetic polymers	Water-soluble	Water-insoluble	Degradable	Small molecule
	HPMC	PVC	Polyanhydride	PE
	Polyacrylate	EVA	PGA	PS
	PEG	PMMA	Polynitrile alkyl acrylate	PEI
	PVA	PE	polyorthoester	PEG
	MC	CA	PHB	
	PAM	PAN	PLLA	
	PEO	PS	PLGA	
	PVP	PPy	PHAS	
		PVDF	PBS	
		EC	PLA	
		PA	PCL	

**Table 3:** Electrospinning combined with other techniques.

Technologies	Drugs	Carrier	Release Mechanism	Highlights	Literature
Hybrid electrospinning + electrospray	FLU/RHB	PLGA	Drug diffusion mechanism Polymer degradation mechanism	The superhydrophobic layer can inhibit the release of FLU and RHB. After 720 h, FLU was released at a rate of about 16.5%, 25.9%, and 37.5%, and RHB was released at a rate of about 21.7%, 29.2%, and 34.6%, respectively, and the deposition times were 5, 10, and 15 min, respectively. It controls the rate of drug release by adjusting the thickness of the superhydrophobic coating.	[214]
Hydrothermal treatment co-precipitation + electrospinning	AMOX	LDH/DMSN/PCL	Diffusion mechanism	The drug release rate of complex membrane A was 87.81%. The drug release rate of complex membrane B was 94.65%.	[215]
Coaxial electrospinning + electrospray	AMPs/Curcumin	PLA/PVP/PEG	Diffusion mechanism	Shell-controlled-release AMPs reached about 70% within 24 h and more than 90% within 72 h for pre-treatment; in the middle and late stages of treatment, the sustained release of curcumin from the core layer can be extended to about 5 days.	[216]
Hybrid electrospinning + solvent steam annealing	SPL	PCL	Diffusion mechanism	Slower SPL release (more than 360 h) can be observed from annealed fibers and a decrease in the final percentage of SPL release (~50–60%).	[217]
Side-by-side electrospinning + electrodeposition	bFGF/NGF	PPy/PVDF	Ion exchange mechanism	Release curves of different growth factors (NGF and bFGF) showed electrically sensitive release behavior, which remained biologically active after release.	[218]
Solution extrusion 3D printing + coaxial electrospinning	Lidocaine/Estradiol/MTZ/CTGF	PCL/PLGA	Drug diffusion mechanism Polymer degradation mechanism	The duration of sustainable release of metronidazole, lidocaine, and estradiol was 4, 25, and 30 days, respectively.	[219]
Hybrid electrospinning + electrospray	CPGs/TRP2/Dox/hpDNA/fBSA	PVA/PEI/PVP/SF	Diffusion mechanism	fBSA and hpDNA were effectively released into the skin, and the cumulative release percentage of DNA was higher than that of BSA.	[220]
Electrospinning + electrospray	CPGs/TRP2/DNA_Trp2_@ETH^MC^/DNA_Trp2_	MC/PVP/HA	Diffusion mechanism	Cumulative transdermal drug release DNA_Trp2_@ETH^MC^ loaded patch within 36 h was 35.4%, significantly higher than the release of free DNA_Trp2_.	[221]
Redox amination + electrospinning	IBU	SA/PVA	Mechanisms of polymer swelling and degradation Diffusion mechanism	Adjusts the drug release rate by adjusting the RAOA/PVA volume ratio. RAOA can effectively encapsulate hydrophobic ibuprofen, thereby slowing the spread rate of the drug.	[222]
Electrospinning + crosslinking post-processing	Dex	SA/PVA	Diffusion mechanism	The release of Dex from the nanofibers was controlled by the chemical potential gradient and expansion penetration. Coaxial nanofibers protected the drug molecule in the core and also supported its sustained release curve.	[223]
Blended electrospinning + casting	IBU	EC/PVP K60	Diffusion mechanism	The first stage exhibited a biphasic controlled release for the pulsating mode, and the residue was released in an extended manner in the second stage.	[224]

## Data Availability

The data supporting the findings of this manuscript are available from the corresponding authors upon reasonable.

## Abbreviations

AgNPs	Ag nano-particles
AMOX	amoxicillin sodium
AMPs	antimicrobial peptides
bFGF	basic fibroblast growth factor
CA	cellulose acetate
CD	cyclodextrin
CIP	ciprofloxacin
Cip HCL	ciprofloxacin hydrochloride
CMC	carboxymethyl cellulose
CMS	carboxymethyl starch
CPGs	CpG motifs
CS	chitosan
CTGF	connective tissue growth factor
DEX	dexamethasone
Dex	dexpanthenol
DMSN	dendritic mesoporous silica nanoparticles
Dox	doxorubicin
DPD	dipyridamole
EC	ethyecellulose
ERS100	Eudragit RS100
ES100	Eudragit S100
ETH^MC^	mannosylated-chitosan (MC) modified Ethosome
EVA	ethyl vinyl acetate
FA	ferulic acid
fBSA	FITC-labeled BSA
FLU	fluorescein
GTP	green tea polyphenol
HA	hyaluronic acid
HPC	hydroxy propyl cellulose
hpDNA	plasmid DNA
HPMC	hydroxypropyl methyl cellulose
IBU	ibuprofen
KET	ketoprofen
LDH	layered double hydroxides
MB	methylene blue
MC	methyl cellulose
MTZ	metronidazole
NGF	nerve growth factor
NR	nature rubber
PAM	polyacrylamide
PAN	polyacrylonitrile
PASP	polyaspartic acid
PBC	probucol
PBS	poly butylenes succinate
PEG	polyethylene glycol
PEI	polyethyleneimine
PEO	polyethylene oxide
PE	polyethylene
PGA	polyglycolic acid
PGlu	polyglutamic acid
PHAS	Poly-hydroxyalkanoates
PHB	polyhydroxybutyrate
PLA	polylactic acid
PLGA	polylacticcoglycollic acid
PLLA	poly-l-lactic acid
PMMA	polymethyl methacrylate
PPy	polypyrrole
PS	polystyrene
PVA	polyvinyl alcohol
PVC	polyvinyl chloride
PVDF	polyvinylidene fluoride
PVP	polyvinylpyrrolidone
RhB	rhodamine B
ROX	roxithromycin
SA	sodium alginate
SF	silk fibroin
SOS	spindles-on-a-string
SPL	spironolactone
TM	timolol maleate
TRP2	tyrosinase-related protein-2
Trp2	tyrosinase-related protein 2
